# Correlation between Quality and Geographical Origins of *Cortex* *Periplocae*, Based on the Qualitative and Quantitative Determination of Chemical Markers Combined with Chemical Pattern Recognition

**DOI:** 10.3390/molecules24193621

**Published:** 2019-10-08

**Authors:** Mengyuan Gao, Xiaohua Jia, Xuhua Huang, Wei Wang, Guangzhe Yao, Yanxu Chang, Huizi Ouyang, Tianxiang Li, Jun He

**Affiliations:** Tianjin State Key Laboratory of Modern Chinese Medicine, Tianjin University of Traditional Chinese Medicine, Tianjin 301617, China; tcmgmy123@163.com (M.G.); jxh2529@163.com (X.J.); xuptz@hotmail.com (X.H.); wwwangwei07@163.com (W.W.); yaoguangzhezy@163.com (G.Y.); tcmcyx@126.com (Y.C.); huihui851025@163.com (H.O.)

**Keywords:** *Cortex Periplocae*, LC-MS/MS, GC-MS, quality assessment, multivariate statistical analysis

## Abstract

Quality assessment of *Cortex Periplocae* remains a challenge, due to its complex chemical profile. This study aims to investigate the chemical components of *Cortex Periplocae*, including its non-volatile and volatile constituents, via liquid chromatograph–mass spectrometry (LC-MS/MS) and gas chromatography–mass spectrometry (GC-MS) assays. The established strategy manifested that *Cortex Periplocae* from different producing areas was determined by identifying 27 chemical markers with ultra-high-performance liquid chromatography, coupled with quadrupole tandem time-of-flight mass spectrometry (UPLC-Q-TOF-MS/MS), including four main groups of cardiac glycosides, organic acids, aldehydes, and oligosaccharides. These groups’ variable importance in the projection (VIP) were greater than 1. Simultaneously, the samples were divided into four categories, combined with multivariate statistical analysis. In addition, in order to further understand the difference in the content of samples from different producing areas, nine chemical markers of *Cortex Periplocae* from 14 different producing areas were determined by high performance liquid chromatography coupled with mass spectrometry (HPLC-MS/MS), and results indicated that the main effective constituents of *Cortex Periplocae* varied with places of origin. Furthermore, in GC-MS analysis, samples were divided into three groups with multivariate statistical analysis; in addition, 22 differential components whose VIP were greater than 1 were identified, which were principally volatile oils and fatty acids. Finally, the relative contents of seven main volatile constituents were obtained, which varied extremely with the producing areas. The results showed that the LC-MS/MS and GC-MS assays, combined with multivariate statistical analysis for *Cortex Periplocae*, provided a comprehensive and effective means for its quality evaluation.

## 1. Introduction

*Cortex Periplocae*, the dried root bark of *Periploca sepium* Bunge, is a perennial liana plant from the Asclepiadaceae family, and it was first recorded in the Sheng Nong’s herbal classic. *Cortex Periplocae* has the effect of diminishing water, swelling, and rheumatism, as well as supporting strong bones, as recorded in the Chinese pharmacopoeia, and it has been widely used for edema, rheumatism, tumor, immunoregulation, and palpitations [1,2,3]. It contains numerous chemical components, such as steroids, cardiac glycosides, terpenoids, and volatile constituents [4,5,6], which confer multiple pharmacological activities. Periplocin, the main bioactive cardiac glycoside in the *Cortex Periplocae*, confers cardiac function effectively, but it can produce gastrointestinal reactions, heart poisoning, liver and kidney toxicity, and even death when one takes it inappropriately [7]. The quality assessment of *Cortex Periplocae* is necessary for medical safety.

A growing number of studies have demonstrated that geographical sources could significantly affect the quality of these herbs, since climate and environment influence biosynthesis and the accumulation of secondary metabolites in organisms [8,9,10]. *Cortex Periplocae* is widespread in Shanxi, Henan, Hebei, and Shandong provinces in China [11,12], and there are no standard planting grounds for it [13]. However, whether or how the quality of *Cortex Periplocae* differs according to its harvested location have not yet been investigated. Such a correlation, if present, would be significant for both a quality assessment and efficient utilization of *Cortex Periplocae*; it could also impact artificial cultivation of *Cortex Periplocae*. Although *Cortex Periplocae* materials from different regions have been chemically analyzed in some studies [14,15], that research aimed to test the feasibility of newly developed analytical assays for *Cortex Periplocae,* and investigations about the correlation between geographical origins and quality were rare. In addition, there was only a single study about the determination of 4-methoxysalicylaldehyde with the HPLC method in Chinese pharmacopoeia in 2015 [1]; past research on quality standards for *Cortex Periplocae* have been mostly about the analysis of periplocin, isovanillin, and 4-methoxysalicylaldehyde. Other compounds have not yet been studied [16,17,18,19,20,21]. As known to all, multi-components in an herb indicate its comprehensive efficacy, and quality assessment based on a few markers has been proven to be insufficient. Therefore, a more systematic and comprehensive method for multicomponent analysis was needed, due to *Cortex Periplocae*’s chemical complexity.

In our study, ultra-high-performance liquid chromatography coupled with quadrupole tandem time-of-flight mass spectrometry (UPLC–Q-TOF-MS/MS) and (HPLC-MS/MS) methods were used for the qualitative analysis and content determination of *Cortex Periplocae*, respectively, as well as gas chromatography–mass spectrometry (GC-MS) for determining volatile constituents, which proved a sensitive and effective method for its quality evaluation. Furthermore, multiple pattern recognition models, including hierarchical cluster analysis (HCA), principal component analysis (PCA), and partial-least-squares discriminate analysis (PLS-DA), were carried out to evaluate the differences in quality of the *Cortex Periplocae* samples. The chemical markers were obtained by the PLS-DA model, which confirmed the reliability of the results. Finally, in order to better understand the differences between geographical sources and quality in terms of content, marker compounds were determined by quantitative analysis. The proposed strategy of LC-MS/MS and GC–MS combined with multivariate statistical analysis can be used to evaluate the quality of *Cortex Periplocae* comprehensively and effectively.

## 2. Results

### 2.1. UPLC-Q-TOF-MS/MS Analysis

**Figure 1:**
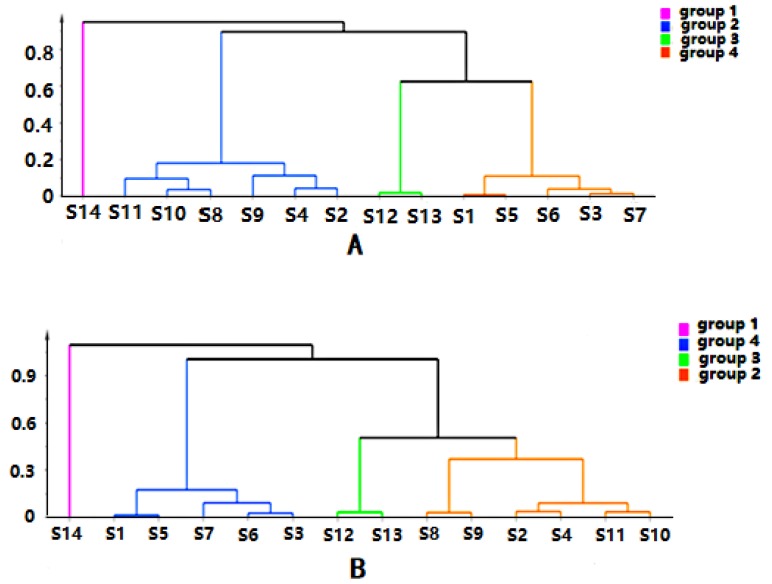
Hierarchical cluster analysis (HCA) of *Cortex Periplocae* from different producing areas, under the mode of positive (**A**) and negative (**B**) ions.

Chromatographic data from 14 batches, collected from UPLC-Q-TOF-MS/MS, were imported into R software and Simca-P 14.1 for multivariate statistical analysis. HCA is mainly used for groups of samples that have not yet been clearly classified [22]. The samples can be categorized intuitively based on the characteristics of their variables. It is an unsupervised pattern recognition method [23]. The HCA diagrams of *Cortex Periplocae* from 14 areas are shown in Figure 1, which indicates that the samples were divided into four categories: S14 (JiXian, Tianjin) was the first category; S2 (FanZhi, Shanxi), S4 (NingWu, Shanxi), S8 (FeiCheng, Shandong), S9 (TaiAn, Shandong), S10 (NanYang, Henan), and S11 (JiaoZuo, Henan) belonged to the second category; S12 (YuXian, Hebei) and S13 (XuanHua, Hebei) belonged to the third category; and the fourth category included S1 (LuCheng, Shanxi), S3 (YuanPing, Shanxi), S5 (YuCi, Shanxi), S6 (LingQiu, Shanxi) and S7 (YangQuan, Shanxi). The model, to some extent, demonstrated a significant relationship between the chemical composition and the source of the samples.

**Figure 2:**
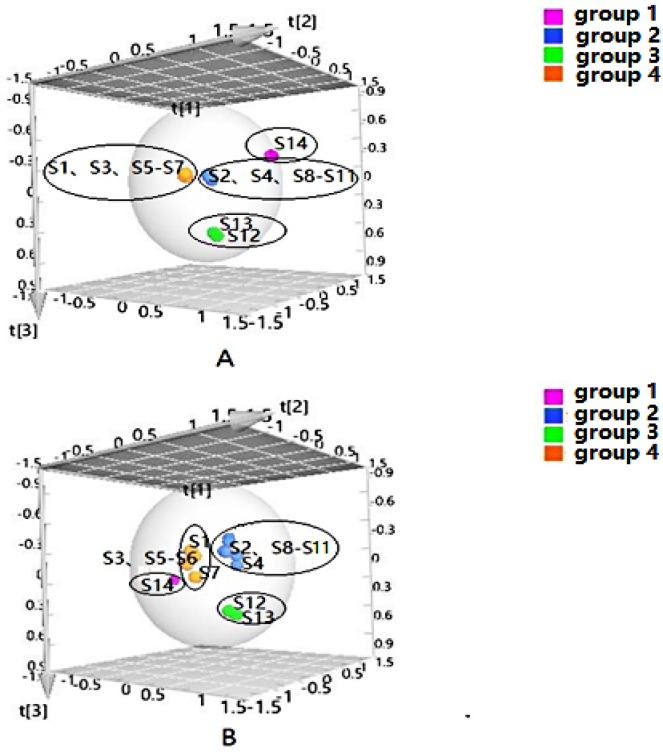
Partial-least-squares discriminate analysis (PLS-DA) of *Cortex Periplocae* from different producing areas, under the mode of positive (**A**) and negative (**B**) ions.

PLS-DA can filter out some random noise, distinguish differences between groups better, and improve the effectiveness and analytical capability of the model. Firstly, PLS-DA analysis was performed on the samples according to the clustering results, as shown in Figure 2. The results displayed that four groups of samples from different habitats were effectively distinguished, and the clustering effect was slightly better than that of HCA. In addition, to screen the chemical markers that contributed more to the differentiation, based on the above HCA and PLS-DA models, the differential components in four groups were analyzed to obtain the variable importance in projection (VIP) values, and compounds with VIP > 1 were used as potential differential components for subsequent qualitative analysis.

**Figure 3:**
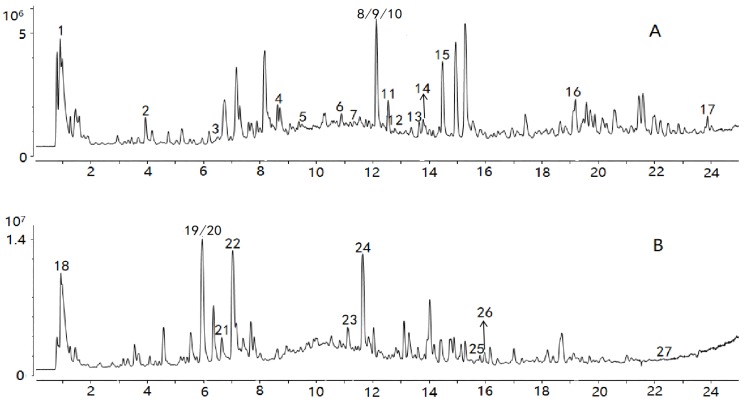
TIC of the sample in positive (**A**) and negative ions (**B**).

Comparisons with reference standards and data in previous reports [24,25,26,27,28,29,30,31,32,33,34,35] enabled tentative identification of 27 constituents whose VIPs were greater than 1, as summarized in Table 1. The spectra of the chemical markers are exhibited in the Supplementary Materials. The 27 constituents are classified into four main groups, including cardiac glycosides, organic acids, aldehydes, and oligosaccharides; they played an important role in differentiation, and were identified as potential chemical markers for quality evaluation of *Cortex Periplocae*. Chlorogenic acid, with VIP = 7.72, differentiated the samples to the biggest extent. Total ion chromatograms (TIC) in positive and negative ions are generated in Figure 3.

### 2.2. HPLC-MS/MS Analysis

#### 2.2.1. Method Validation

Quantitative method validation for the established HPLC-MS/MS analysis was performed for linearity, lower limits of detection (LLODs), lower limits of quantification (LLOQs), intra- and inter-day precision, repeatability, stability, and recovery. The results are shown in Table 2 and Table 3. All correlation coefficient values (*r* ≥ 0.9990) demonstrated a good linear relationship between the analyte concentrations and their peak areas within the relatively wide test ranges. The LLOD and LLOQ were determined at a signal-to-noise (S/N) ratio of approximately 3 and 10, respectively, and the results showed that the LLODs and LLOQs of the analytes were in the range of 0.1–5.0 ng/mL and 0.3–10.0 ng/mL, respectively. The intra- and inter-day precisions of nine analytes (Rlative Sandard Deviation—RSDs) were within 0.4%–2.7% and 0.3%–5.3%, respectively. The RSDs for repeatability were less than 4.2%. As for stability, the RSDs were lower than 4.9%. The developed method also had acceptable accuracy, with spike recovery of 89.4%–105.9% for all analytes. The results revealed that the established method was precise enough for simultaneous quantitative determination of the nine components.

#### 2.2.2. Determination of Nine Components

**Figure 4:**
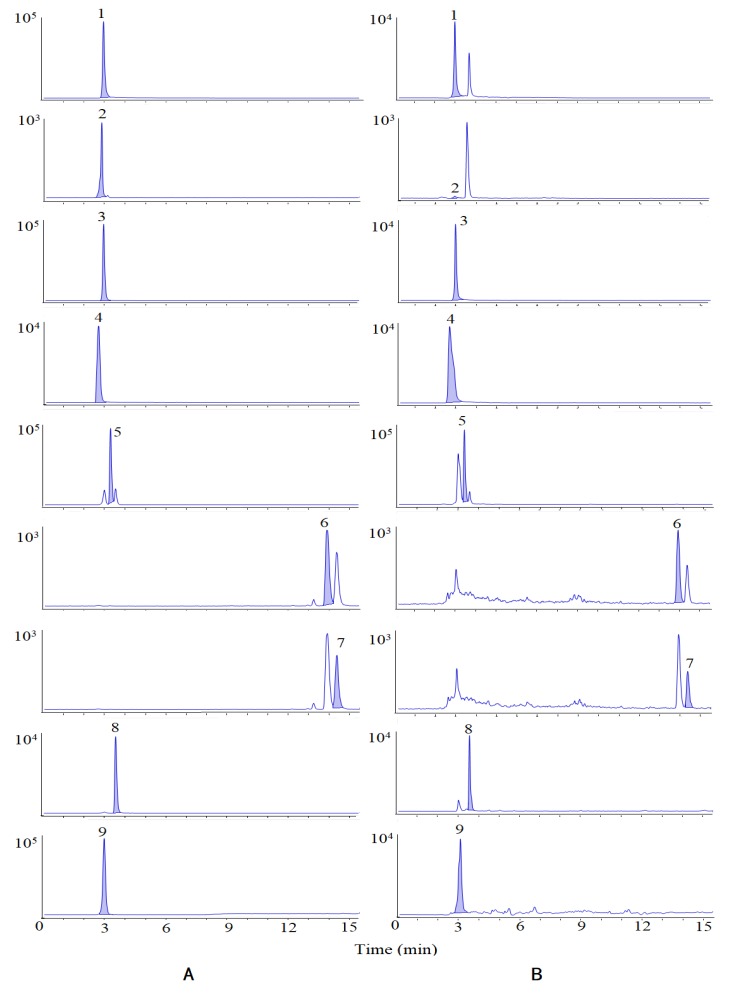
MRM of standards (**A**) and samples (**B**). Peaks are as follows: (1) isovanillin, (2) isovanillic acid, (3) scopoletin, (4) chlorogenic acid, (5) periplogenin, (6) oleanolic acid, (7) ursolic acid, (8) periplocymarin, and (9) periplocin.

Based on the UPLC-Q-TOF-MS/MS analysis, 27 compounds whose VIP were greater than 1 were identified as the chemical markers. In order to further understand the variations of the samples from different content levels, nine bioactive chemical markers were determined by HPLC-MS/MS. The Multiple Reaction Monitoring (MRM) diagram is shown in Figure 4.

The concentrations of nine compounds were recorded and the contents were calculated. The results (Table 4) reveal that the contents of the nine constituents varied with their origins. These quantitative results of nine bioactive chemical markers provide a valuable reference for differentiating samples collected from different geographical regions.

### 2.3. Qualitative Analysis Based on GC-MS

#### 2.3.1. Optimization of GC-MS Conditions

The DB-5 and DB-17 columns were both used for chromatographic separation. Results of the DB-17 column showed better peak shape and had much greater peaks, as compared to the DB-5 column. In addition, the shunt ratio was mainly used for macroanalysis or analysis of non-dilutable samples. In this experiment, shunt ratios of 10, 20, 30, and 50 were selected for analysis, and it was observed that shunt ratio of 30 provided better peak shapes compared to the rest.

#### 2.3.2. Sample Analysis

At present, there are few studies on the volatile components of *Cortex Periplocae*, and reports on the quantitative analysis of such active ingredients are rare. Therefore, the GC-MS method was established for further understanding of the diversities of the samples from different origins.

**Figure 5:**
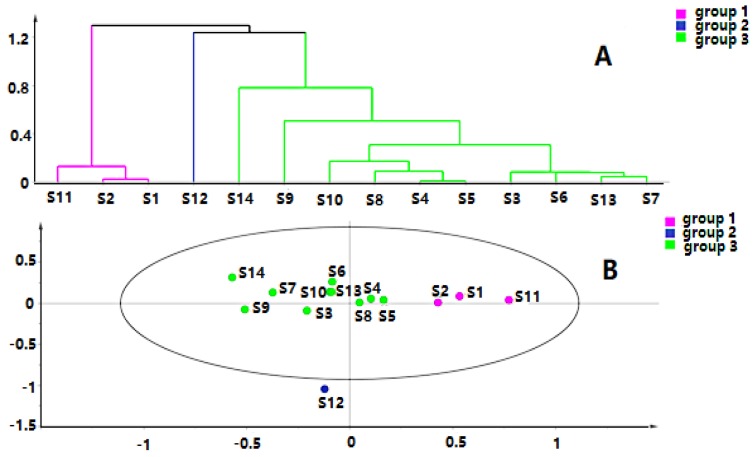
HCA (**A**) and principal component analysis (PCA) (**B**) of *Cortex Periplocae* from different producing areas.

**Figure 6:**
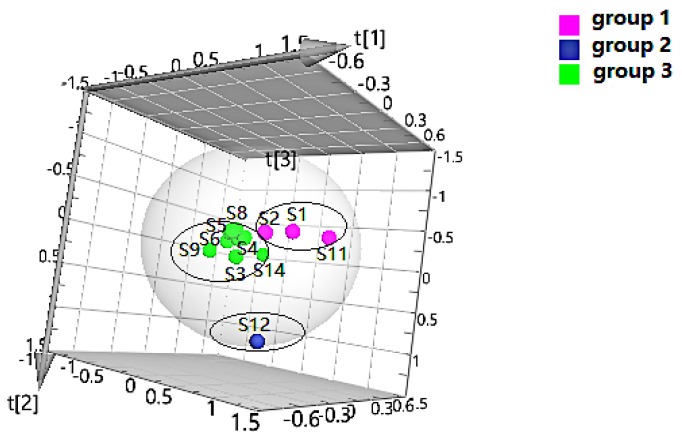
PLS-DA of *Cortex Periplocae* from different producing areas.

**Figure 7:**
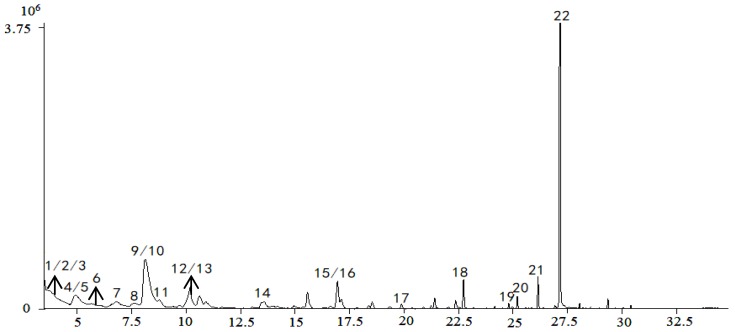
TIC of the sample, based on gas chromatography–mass spectrometry (GC-MS).

The quality data collected from GC-MS were converted into the MZ DATA format, and were then imported into R software and Simca-P 14.1 for multivariate statistical analysis. The HCA and PCA diagrams of *Cortex Periplocae* from 14 areas are shown in Figure 5. It shows that the samples were divided into three categories: S1 (LuCheng), S2 (FanZhi), and S11 (JiaoZuo) are in the first category; S12 (YuXian) belongs to the second category; and S3–S10 (YuanPing, NingWu, YuCi, LingQiu, YangQuan, FeiCheng, TaiAn, NanYang, respectively), as well as S13 (XuanHua) and S14 (JiXian) belong to the third cateogry. The results of the two models verified each other. Furthermore, PLS-DA was also performed for better improvement of model effectiveness, as shown in Figure 6. The samples were also divided into three groups, like the HCA and PCA model. The results proved that the volatile constituents varied greatly with herbal origins. In addition, based on the above models, the differential volatile components in the three groups were analyzed to obtain the table of VIP values, and compounds with VIP > 1 were used as potential volatile chemical markers for subsequent qualitative analysis. The TIC diagrams of the *Cortex Periplocae* sample was exhibited in Figure 7.

Twenty-two total volatile compounds with VIP > 1 were tentatively identified from different origins, based on the accurate relative molecular mass and the National Institute of Standards and Technology (NIST) 08 mass spectral library. The 22 volatile constituents, as listed in Table 5, were principally volatile oils and fatty acids, and they were identified as potential volatile chemical markers for the quality assessment of *Cortex Periplocae*.

### 2.4. Relative Content Analysis Based on GC-MS

The volatile oil, one of the active components of *Cortex Periplocae*, confers insecticidal activity effectively [36]. Among the 22 volatile chemical markers, seven constituents presented a higher concentration compared to the others, which were determined so as to further understand the differences in volatile components at the content level. A quantitative analysis in percent was determined by peak area normalization. The results were calculated by GC-MS Postrun Analysis software, and summarized in Table 6. The results revealed that the content of volatile constituents did vary greatly with the geographical locations.

## 3. Materials and Methods

### 3.1. Sample Collection

Fourteen batches of *Cortex Periplocae* excavated in the spring were collected from five different provinces (Shanxi, Shandong, Hebei, Henan, and Tianjin) of China. They were identified as the dried bark of *Cortex Periplocae* by Professor Li Tianxiang and stored in Tianjin University of Traditional Chinese Medicine; the sample information is shown in Table 7.

### 3.2. UPLC-Q-TOF-MS/MS Analysis

#### 3.2.1. Chemicals and Apparatus

Periplocin (≥98%), periplocymarin (≥98%), periplogenin (≥98%), and scopoletin (≥98%) were purchased from Chengdu Durst Biotechnology Co., Ltd. (Chengdu, China). Isovanillin (≥98%), isovanillic acid (≥98%), chlorogenic acid (≥98%), ursolic acid (≥98%), and oleanolic acid (≥98%) were obtained from Shanghai Yuanye biotechnology Co., Ltd. (Shanghai, China). Chromatographic grade methanol and acetonitrile were purchased from Fisher company (Thermo Fisher Scientific (China) Co. Ltd., Shanghai, China). Anhydrous ethanol (chromatographically pure) was obtained from Concord technologies Ltd. (Tianjin, China). Water was obtained from a Milli-Q purification system (Millipore, Bedford, MA, USA). Nylon membranes were purchased from Tianjin Bojin Technology Co., Ltd. (Tianjin, China).

The UPLC-Q-TOF-MS/MS system is comprised of an Agilent 1290 Infinity UPLC (Agilent Technologies Inc., Palo Alto, CA, USA) and an Agilent 6520 QTOF. The UPLC system includes an Agilent 4220 binary pump combined with a degasser, an Agilent 4212 diode array detector, an Agilent 4226 well plate sampler, and an Agilent 1316 thermostatic column compartment. Chromatographic separation was performed on a Waters UPLC^®^BEH C18 column (2.1 × 100 mm, 1.7 µm, Waters, Milford, MA, USA).

#### 3.2.2. Sample Preparation and Measurement

All the samples were dried for 4 h at 60 °C, grounded, and passed through a 50-mesh sieve. Samples of 0.5 g were immersed in 5 mL of 60% methanol and then ultrasonicated for 30 min. The solution was centrifuged for 10 min at 14000 r∙min^−1^, and the resulting supernatant was filtered with 0.22 µm nylon membranes for analysis. Primary stock solutions of nine reference compounds were a mixture of different concentration of standards in methanol.

The mobile phase consisted of 0.1% formic acid (solvent A) and acetonitrile (solvent B), with a gradient elution program of 3%–10% B at 0–5 min, 10%–30% B at 5–10 min, 30%–60% B at 10–16 min, and 60%–99% B at 16–25 min, in a positive ion pattern; and 3%–18% B at 0–7 min, 18%–62% B at 7–16 min, and 62%–99% B at 16–25 min in a negative ion pattern. The flow rate was 0.3 mL/min and the injection volume was 1 µL. The optimized conditions were as follows: dry gas temperature at 350 °C, dry gas flow rate at 9 L/min, nebulizer pressure at 30 psi, skimmer at 65 V, capillary voltage at 3500 V, sheath gas temperature at 300 °C, collision energy at 35 V, and fragmentor voltage at 175 V. Mass spectra were recorded across the range of *m*/*z* 50–1500.

#### 3.2.3. Data Pre-Processing

Firstly, the collected data were converted into MZ Data format by Agilent Masshunter analysis software, and then peak extraction, peak alignment, and peak matching were carried out by R software to obtain the retention time (Rt), mass-to-charge ratio (*m*/*z*), and peak strength of each compound. Then the missing values were removed, according to the 80% modified principle. Finally, the obtained data were imported into Simca-P 14.1 for multivariate statistical analysis to screen for VIP > 1 compounds as potential chemical markers.

### 3.3. HPLC-MS/MS Analysis

#### 3.3.1. Chemicals and Apparatus

Chromatographic analysis was performed on an Agilent 1200 HPLC system (Agilent Technologies Inc., Palo Alto, CA, USA) equipped with an Agilent 6430 Triple quadrupole tandem mass spectrometer. A CORTECS C18 column (4.6 × 150 mm, 2.7 µm; Waters, Milford, MA, USA) was used for the chromatographic separation, and the temperature of 15 °C was maintained. The chemicals were the same as those used for the UPLC-Q-TOF-MS/MS analysis.

#### 3.3.2. Sample Preparation and Measurement

The mobile phase consisted of 0.1% formic acid (solvent A) and methanol (solvent B), and the gradient elution for HPLC-MS/MS analysis was performed as follows: 82%–82% B at 0.0–3.6 min, 82.0%–92.5% B at 3.6–3.7 min, and 92.5%–92.5% B at 3.7–15.5 min. The flow rate was 0.5 mL/min, and the injection volume was 5 µL. The mass spectrometry was acquired in positive ion and negative ion modes. The instrumental parameters were as follows: gas temperature at 300 °C, gas flow rate at 11 L/min, nebulizer pressure at 15 psi, and delta Electron multiplier voltage (EMV) (±) at 500 V. Mass spectral parameters and ion patterns are shown in Table 8. The sample preparation was the same as described in Section 3.2.2.

### 3.4. GC-MS Analysis

#### 3.4.1. Apparatus

The volatile components were analyzed by a Shimadzu QP 2010 GC-MS, equipped with a DANI Hss 86.50 headspace sampler and AOC-20i Autosampler. Chromatographic separation was performed on a DB-17 column (0.25 mm × 30 m × 0.25 µm).

#### 3.4.2. Sample Preparation and Measurement

All the samples were dried for 4 h, ground, passed through a 50-mesh sieve, and sealed in a headspace bottle for analysis.

The temperature conditions used were as follows: 50 °C for 0 min, 50–110 °C at 3 °C/min, 110–200 °C at 8 °C/min, and held constant for 6 min. Ionization was performed in the electron impact mode at 70 eV. The ion source temperature and the interface temperature were 230 °C and 250 °C, respectively. Electron Impact (EI) spectra were recorded in full scan mode at *m*/*z* 33–500.

#### 3.4.3. Data Pre-Processing

The collected qualitative data were converted into MZ Data format by GC-MS Postrun Analysis software first, then R software and Simca-P 14.1 software were used for further analysis in the processing of UPLC-Q-TOF-MS/MS data.

## 4. Conclusions

In this experiment, UPLC-Q-TOF-MS/MS and HS-GC-MS methods combined with multivariate statistical analysis technology were applied to screen differential chemical compositions of *Cortex Periplocae* from different origins. According to the multivariate statistical analysis for both volatile and nonvolatile components, the samples were clearly classified, suggesting that these categories should be addressed in the preparation of products. Based on PLS-DA model, 49 chemical markers with VIP > 1 were identified, which was of great significance to distinguish the samples from different origins. Furthermore, HPLC-MS/MS was used for quantitative analysis of nine effective chemical components of *Cortex Periplocae* from 14 places of origin. The relative contents of seven volatile chemical markers with higher concentrations were determined by GC-MS. The results indicated that the content of active ingredients varied greatly from place to place. In conclusion, the established strategy based on LC-MS/MS and GC-MS, combined with multivariate statistical analysis, was carried out to in order to clarify the relationship between the quality and geographical origin of *Cortex Periplocae*, and it would be an efficient and applicable tool for the quality assessment of *Cortex Periplocae*.

## Figures and Tables

**Table 1:** Chemical markers identified, based on UPLC-Q-TOF-MS/MS.

Number	Ion Mode	Precursor Ion	Fragment Ion	Retention Time (min)	Loading Form	Possible Compound	Molecular Formula	Ref
1	ESI(+)	365.1045	85.0002	1.136	[M + Na]^+^	Melibiose	C_12_H_22_O_11_	[24]
2	ESI(+)	343.1361	163.0608	3.847	[M + H]^+^	Citrusin D	C_16_H_22_O_8_	[25]
3	ESI(+)	265.1420	247.1310, 211.1034, 183.1007, 173.0083	6.447	[M + H]^+^	Ridentin	C_15_H_20_O_4_	[26]
4	ESI(+)	153.0539	61.0601, 121.0209	8.726	[M + H]^+^	Isovanillin	C_8_H_8_O_3_	*
5	ESI(+)	193.0489	178.0268, 137.0578, 122.0957	9.497	[M + H]^+^	Scopoletin	C_10_H_8_O_4_	*
6	ESI(+)	517.1327	499.1221, 355.1088, 163.0389	10.808	[M + H]^+^	Isochlorogenic Acid B	C_25_H_24_O_12_	[27]
7	ESI(+)	339.1063	257.1124, 89.0595	11.367	[M + Na]^+^	Vanilloloside	C_14_H_20_O_8_	[28]
8	ESI(+)	391.2473	355.2254, 337.2148	12.084	[M + H]^+^	Periplogenin	C_23_H_34_O_5_	*
9	ESI(+)	391.2471	373.2366, 275.1125	12.164	[M + H]^+^	Tussilagone	C_23_H_34_O_5_	[29]
10	ESI(+)	169.0483	151.0381, 109.1011	12.231	[M + H]^+^	Isovanillic Acid	C_8_H_8_O_4_	*
11	ESI(+)	719.3596	391.2473, 373.2358, 355.2255, 337.2145	12.543	[M + Na]^+^	Periplocin	C_36_H_56_O_13_	*
12	ESI(+)	413.2287	391.2476, 373.2363, 355.2259, 337.2153	12.748	[M + Na]^+^	Periplogenin 1	C_23_H_34_O_5_	[30]
13	ESI(+)	487.2996	339.1085	13.709	[M + Na]^+^	Periplocin N	C_27_H_44_O_6_	[30]
14	ESI(+)	557.3058	391.2457, 373.2364, 355.2243, 337.2136	13.965	[M + Na]^+^	Periplocymarin	C_30_H_46_O_8_	*
15	ESI(+)	327.1941	675.3627, 349.1768	14.322	[M + H]^+^	12β-Hydroxyl progesterone-4,6,13-triene-3,20-diketone	C_21_H_26_O_3_	[30]
16	ESI(+)	279.2317	261.2201, 121.0509	19.132	[M + H]^+^	Linolenic Acid	C_18_H_30_O_2_	[31]
17	ESI(+)	457.3639	439.3556, 249.1764	24.179	[M + H]^+^	Oleanolic Acid	C_30_H_48_O_3_	*
18	ESI(−)	377.0893	341.1124, 179.0589, 683.2303	1.012	[M + Cl]^−^	Saccharose	C_12_H_22_O_11_	[30]
19	ESI(−)	191.0571	161.0244	5.894	[M−H]^−^	Quinic Acid	C_7_H_12_O_6_	[32]
20	ESI(−)	353.0913	191.0586	5.896	[M − H]^−^	Chlorogenic Acid	C_16_H_18_O_9_	*
21	ESI(−)	355.1069	175.0513, 149. 0372	6.726	[M − H]^−^	Gentiopicrin	C_16_H_20_O_9_	[33]
22	ESI(−)	581.2290	419.1728	7.207	[M − H]^−^	5,5’-Dimethoxylariciresil 4-*O*-glucoside	C_28_H_38_O_13_	[30]
23	ESI(−)	687.3643	641.3563	11.375	[M+COOH]^−^	Periplocin C	C_33_H_54_O_12_	[30]
24	ESI(−)	167.033	108.0202, 123.0434	11.729	[M − H]^−^	4-Methoxysalicylic acid	C_8_H_8_O_4_	[30]
25	ESI(−)	487.3468	96.9627	15.563	[M − H]^−^	Arjunolic Acid	C_30_H_47_O_5_	[34]
26	ESI(−)	485.3311	449.2348, 327.2205	15.639	[M − H]^−^	24-hydroxyglycyrrhetic acid	C_30_H_46_O_5_	[35]
27	ESI(−)	455.3566	325.1872, 186.1062	22.347	[M − H]^−^	Ursolic Acid	C_30_H_48_O_3_	*

ESI: Electrospray Ionization *: the compound was identified by the standard.

**Table 2:** Standard curve regression equation, lower limit of detection (LLOD), and lower limit of quantification (LLOQ) of nine components.

Compound	Linear Equation	*r*	Linearity Range	LLOD	LLOQ
			(ng mL^−1^)	(ng mL^−1^)	(ng mL^−1^)
isovanillin	y = 75.125x + 8936.830	0.9997	25–10,000	2	5
isovanillic acid	y = 5.177x + 92.148	0.9998	5–2000	1	2.5
scopoletin	y = 86.980x + 6320.950	0.9996	25–10,000	0.5	1
chlorogenic acid	y = 36.114x + 2307.730	0.9995	25–10,000	2	5
periplogenin	y = 70.338x + 3893.889	0.9997	30–12000	0.5	1
oleanolic acid	y = 14.851x + 5409.933	0.9990	25–10,000	2	5
ursolic acid	y = 18.160x + 2493.427	0.9991	12.5–5000	2	5
periplocymarin	y = 23.597x + 951.945	0.9998	25–10,000	0.1	0.3
periplocin	y = 141.294x + 3372.552	0.9996	25–10,000	5	10

**Table 3:** Precision, repeatability, stability, and recovery of nine analytes.

Compound	Precision (RSD, %)		Repeatability (RSD, %)	Stability (RSD, %)	Sample Recovery	
	Intra-Day	Inter-Day			Average Recovery Rate (%)	RSD (%)
isovanillin	0.4	4.0	1.2	1.8	96.5	4.3
isovanillic acid	2.4	1.7	2.8	2.9	99.6	3.1
scopoletin	1.0	1.5	1.4	0.8	101.4	2.0
chlorogenic acid	2.1	1.9	2.0	3.6	100.9	4.1
periplogenin	0.4	0.3	3.1	3.2	89.9	2.8
oleanolic acid	2.0	5.3	1.2	4.4	105.9	2.3
ursolic acid	0.7	4.5	4.2	4.9	89.4	3.7
periplocymarin	1.3	2.3	1.7	2.3	99.9	1.5
periplocin	2.7	3.9	1.2	3.5	98.0	0.9

**Table 4:** The contents of nine components in *Cortex Periplocae* from different areas (μg·g^−1^).

Sample	Isovanillin	Isovanillic Acid	Scopoletin	Chlorogenic Acid	Periplogenin	Oleanolic Acid	Ursolic Acid	Periplocymarin	Periplocin
S1	26.59	-	8.81	74.41	2.46	16.91	4.86	2.22	9.08
S2	62.53	1.52	12.42	41.38	2.13	9.47	2.15	3.66	19.60
S3	17.45	-	2.50	43.41	1.28	0.53	1.38	0.54	7.36
S4	46.01	1.54	6.53	45.83	8.39	2.08	0.30	5.95	30.57
S5	31.12	-	4.14	52.19	3.42	0.67	0.17	0.89	10.29
S6	37.63	-	25.64	69.85	5.69	27.95	7.03	2.62	18.32
S7	26.34	-	6.24	46.48	4.98	26.47	7.13	1.14	10.15
S8	31.56	19.40	19.97	43.15	0.67	23.29	13.51	0.74	1.27
S9	46.75	0.94	30.75	37.79	5.16	31.65	14.95	7.65	10.75
S10	37.70	-	14.79	44.18	1.54	11.11	6.21	0.58	10.15
S11	99.07	-	36.34	27.86	2.19	29.42	13.89	2.50	6.48
S12	17.79	0.45	3.22	49.68	21.57	7.35	1.34	4.63	31.78
S13	17.44	-	1.13	50.57	21.17	0.14	0.21	3.75	43.81
S14	25.37	2.04	31.65	13.41	96.60	49.85	22.62	40.04	31.57

**Table 5:** Chemical markers identified, based on GC-MS.

NO.	Retention Time (min)	Compound	Molecular Mass	Molecular Formula	Retention Index
1	3.755	6-Methyl-2-heptanone	128.212	C_8_H_16_O	888
2	3.79	4-Hydroxynicotinic acid	139.109	C_6_H_5_NO_3_	1364
3	3.9	3, 4-Dimethyl-2-cyclopenten-1-one	110.154	C_7_H_10_O	904
4	4.89	2-Methylpiperazine	100.162	C_5_H_12_N_2_	1072
5	4.95	Hexanal	100.159	C_6_H_12_O	806
6	5.61	oct-3-yn-2-one	124.180	C_8_H_12_O	970
7	6.455	2-Ethyl-4-methyl-1-pentanol	130.228	C_8_H_18_O	931
8	7.49	trans cis-1,2,4-trimethy clohexane	126.239	C_9_H_18_	903
9	8	2,3-Dimethylphenol	122.164	C_8_H_10_O	1127
10	8.035	2,7-dimethyloxepine	122.164	C_8_H_10_O	954
11	8.67	(±)-1-phenylethanol	122.164	C_8_H_10_O	1055
12	10.045	2-Amylfuran	138.207	C_9_H_14_O	1040
13	10.445	4-methylcyclohexanol acetate	156.222	C_9_H_16_O_2_	1108
14	13.475	2-ethyl-2-hexenal	126.196	C_8_H_14_O	990
15	16.825	Tetrahydrofurfurylamine	101.147	C_5_H_11_NO	893
16	17.06	Phenylacetaldehyde	120.148	C_8_H_8_O	1081
17	19.755	2,6,6-trimethylcyclohepta-2,4-dien-1-one	150.218	C_10_H_14_O	1199
18	22.67	phenylthiocyanate	135.186	C_7_H_5_NS	1290
19	24.73	1,3-Dimethoxy-5-methylbenzene	152.190	C_9_H_12_O_2_	1172
20	25.12	Safrole	162.185	C_10_H_10_O_2_	1345
21	26.075	4-Hydroxy-3-methoxystyrene	150.174	C_9_H_10_O_2_	1293
22	27.04	4-methoxysalicylaldehyde	152.150	C_8_H_8_O_3_	1392

**Table 6:** The relative content of volatile components in *Cortex Periplocae* from different habitats (%).

NO.	Compound	S1	S2	S3	S4	S5	S6	S7	S8	S9	S10	S11	S12	S13	S14
1	2,3-Dimethylphenol	17.84	14.3	24.81	16.63	15.05	26.09	23.11	11.76	10.08	10.79	15.14	-	18.08	37.52
2	2-Amylfuran	2.02	2.13	2.22	3.43	2.19	3.48	2.65	8.1	8.48	7.6	1.62	-	4.08	10.06
3	2-ethyl-2-hexenal	-	0.37	-	0.29	-	0.75	0.21	0.3	0.96	1.17	1.31	1.28	0.24	0.63
4	2, 6, 6-trimethyl-cyclohepta-2, 4-dien-1-one	0.58	1.78	1.13	2.35	1.55	1.29	0.94	1.18	2.05	0.18	0.39	0.59	1.55	-
5	1, 3-Dimethoxy-5-methylbenzene	0.29	0.99	0.29	1.29	1.02	1.04	0.59	0.73	1.31	0.03	0.21	0.33	0.82	0.02
6	Safrole	0.63	2.63	0.78	3.13	2.08	2.83	1.43	1.75	2.72	0.11	0.5	0.71	1.7	0.71
7	4-methoxysalicylaldehyde	51.2	44.14	28.77	34.58	51.2	30.72	26.03	35.4	21.42	32.25	54.6	28.6	31.93	22.53

**Table 7:** Sample information of *Cortex Periplocae.*

Sample	Origin			Harvest Time
S1	Shanxi	Changzhi City	Lucheng	20160422
S2		Xinzhou City	Fanzhi	20160425
S3		Xinzhou City	Yuanping	20160424
S4		Xinzhou City	Ningwu	20160424
S5		Jinzhong City	Yuci	20160430
S6		Datong City	Lingqiu	20160426
S7		Yangquan City		20160422
S8	Shandong	Linyi City	Feixian	20160416
S9		Taian City		20160417
S10	Henan	Nanyang City		20160413
S11		Jiaozuo City		20160415
S12	Hebei	Zhangjiakou CIity	Yuxian	20160501
S13		Zhangjiakou CIity	Xuanhua	20160430
S14	Tianjin	Jixian		20160509

**Table 8:** Mass spectrometry parameters of nine target compounds.

Compound	Ion Mode	Precursor Ion (*m*/*z*)	Product Ion (*m*/*z*)	Fragmentor (V)	Collision Energy (V)
isovanillin	Positive	153.0	65.1	105	23
isovanillic acid	Positive	169.0	65.0	115	26
scopoletin	Positive	193.0	133.0	110	20
periplogenin	Positive	391.3	337.2	135	10
periplocymarin	Positive	535.3	113.1	135	20
periplocin	Positive	719.4	719.4	135	0
chlorogenic acid	Negative	353.0	191.1	90	10
oleanolic acid	Negative	455.2	455.2	145	0
ursolic acid	Negative	455.2	455.2	145	0

## Supplementary Materials

The supplementary materials are available online.
