# Supplementary material for: Correlation between Quality and Geographical Origins of Cortex Periplocae, Based on the Qualitative and Quantitative Determination of Chemical Markers Combined with Chemical Pattern Recognition
Source: Molecules. 2019 Oct 8;24(19):3621. doi: 10.3390/molecules24193621 (PMC6804018; doi:10.3390/molecules24193621)
Supplement: Supplementary file 1 [file molecules-24-03621-s001.pdf]

Supplementary Materials

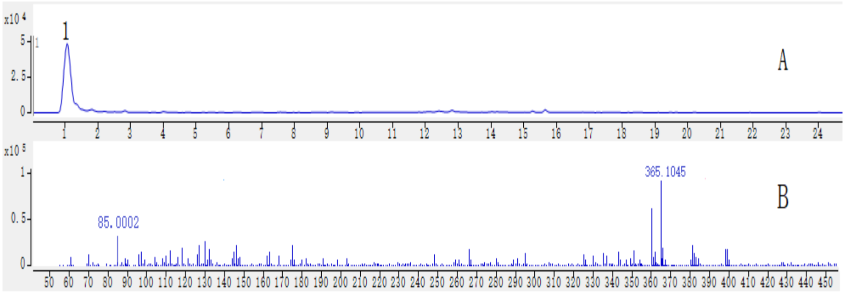

Spectra of Melibiose (1). A: extraction ion chromatography; B: mass spectra in high CE mode.

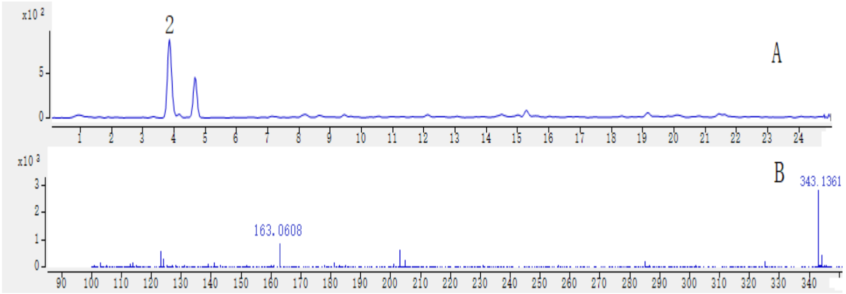

Spectra of Citrusin D (2). A: extraction ion chromatography; B: mass spectra in high CE mode.

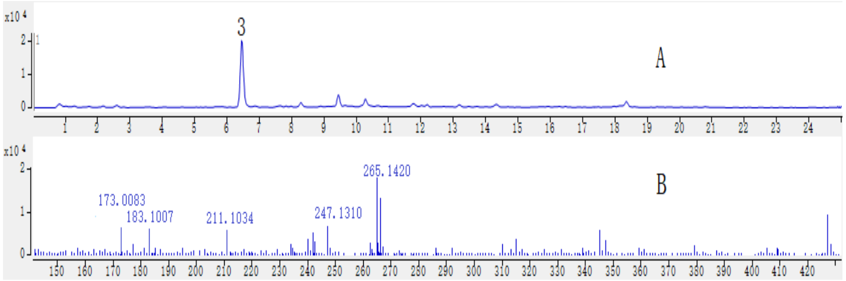

Spectra of Ridentin (3). A: extraction ion chromatography; B: mass spectra in high CE mode.

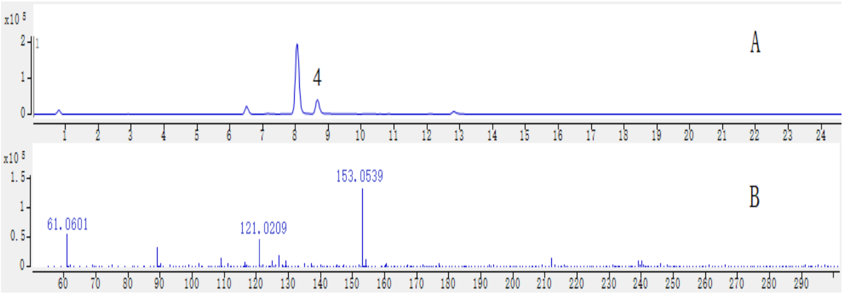

Spectra of Isovanillin (4). A: extraction ion chromatography; B: mass spectra in high CE mode.

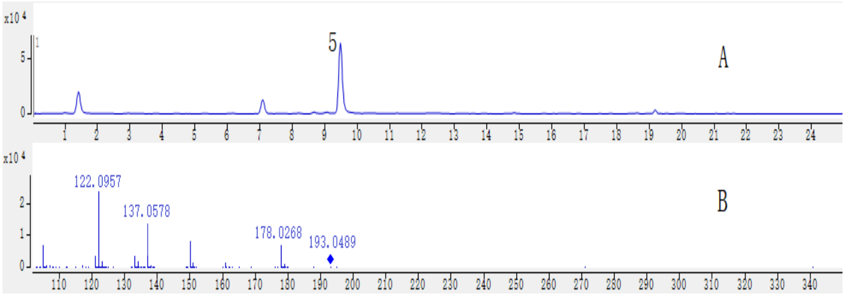

Spectra of Scopoletin (5). A: extraction ion chromatography; B: mass spectra in high CE mode.

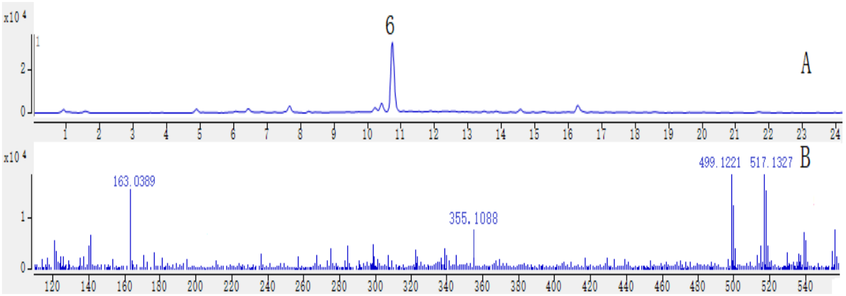

Spectra of Isochlorogenic Acid B (6). A: extraction ion chromatography; B: mass spectra in high CE mode.

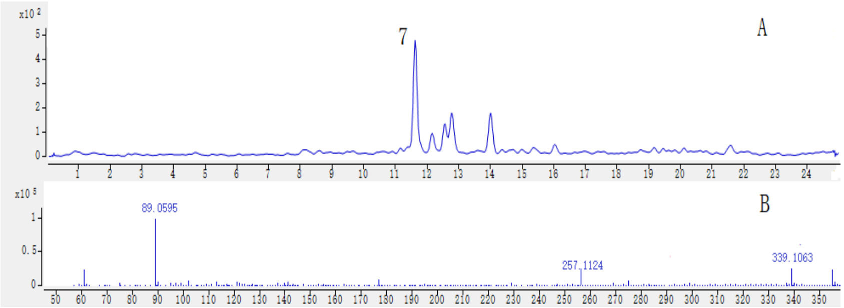

Spectra of Vanilloloside (7). A: extraction ion chromatography; B: mass spectra in high CE mode.

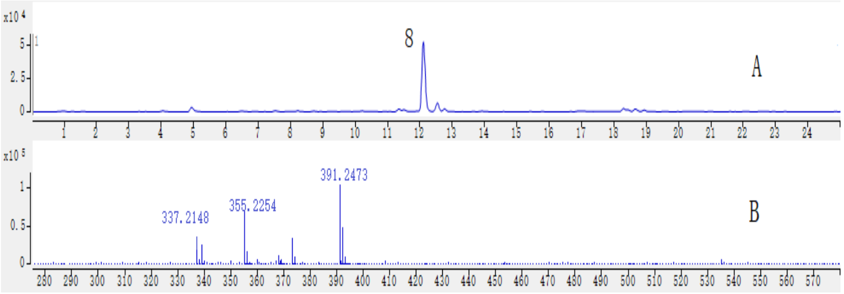

Spectra of Periplogenin (8). A: extraction ion chromatography; B: mass spectra in high CE mode.

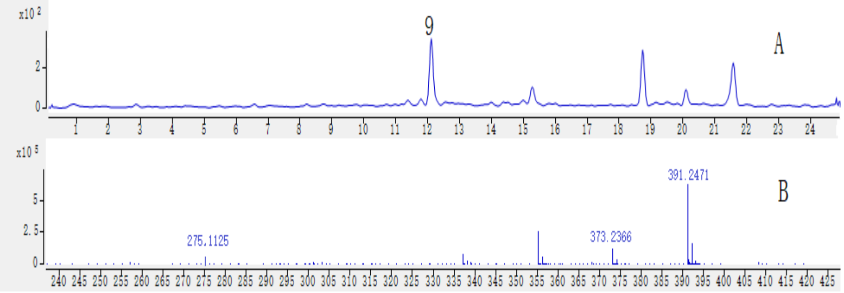

Spectra of Tussilagone (9). A: extraction ion chromatography; B: mass spectra in high CE mode.

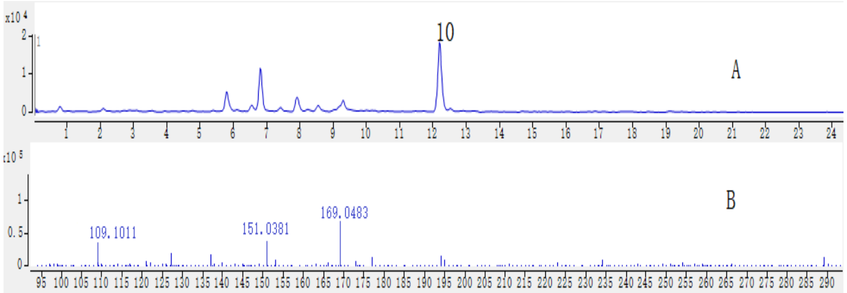

Spectra of Isovanillic Acid (10). A: extraction ion chromatography; B: mass spectra in high CE mode.

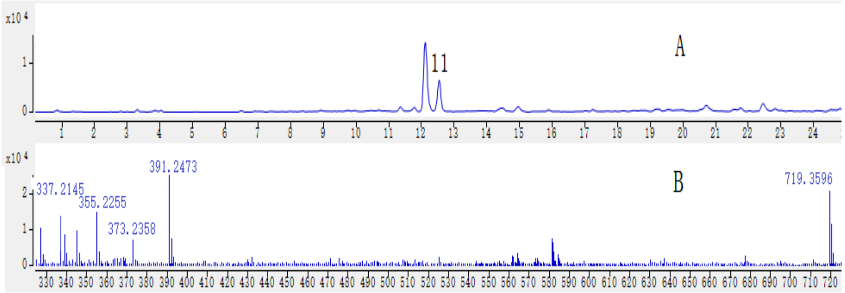

Spectra of Periplocin (11). A: extraction ion chromatography; B: mass spectra in high CE mode.

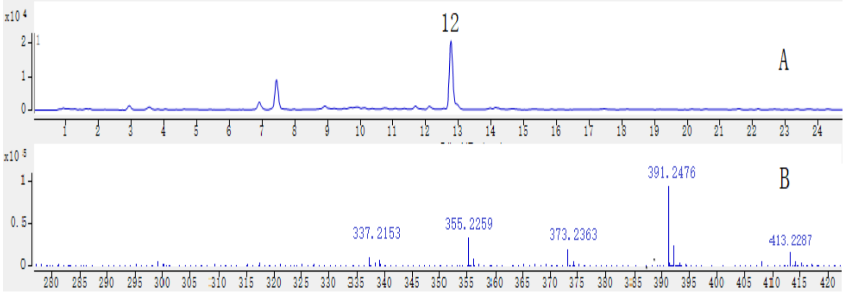

Spectra of Periplogenin 1 (12). A: extraction ion chromatography; B: mass spectra in high CE mode.

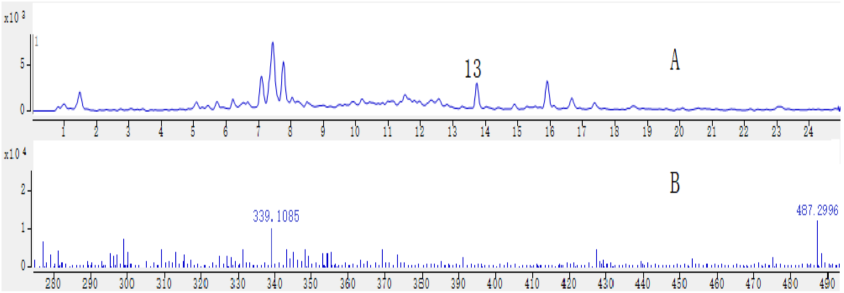

Spectra of Periplocin N (13). A: extraction ion chromatography; B: mass spectra in high CE mode.

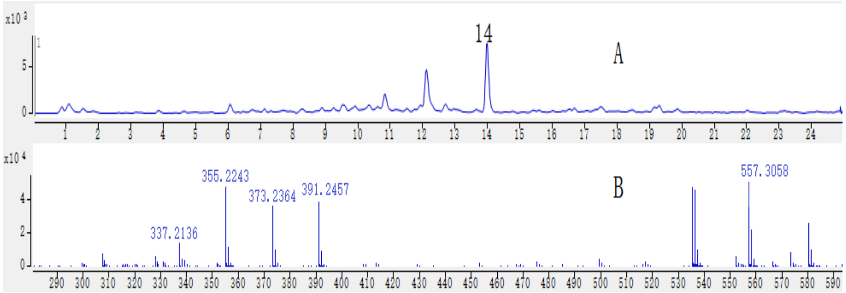

Spectra of Periplocymarin (14). A: extraction ion chromatography; B: mass spectra in high CE mode.

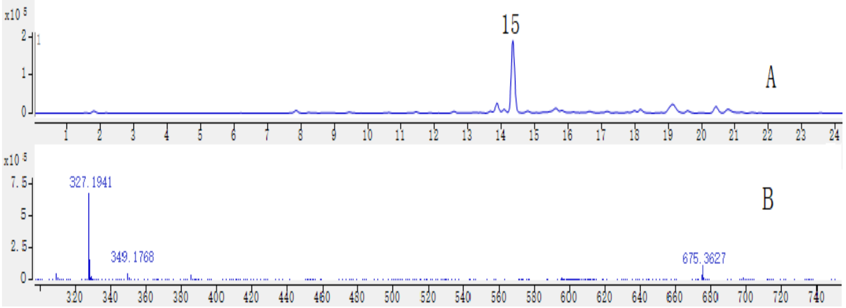

Spectra of 12β-Hydroxyl progesterone-4,6,13-triene-3,20-diketone (15). A: extraction ion chromatography; B: mass spectra in high CE mode.

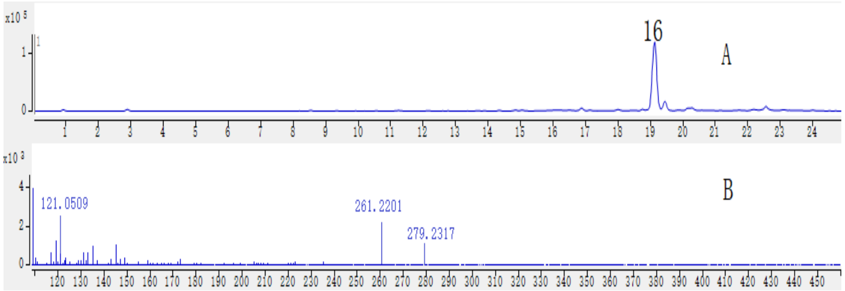

Spectra of Linolenic Acid (16). A: extraction ion chromatography; B: mass spectra in high CE mode.

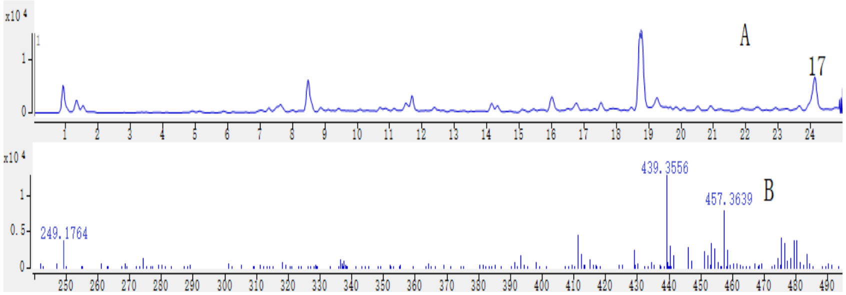

Spectra of Oleanolic Acid (17). A: extraction ion chromatography; B: mass spectra in high CE mode.

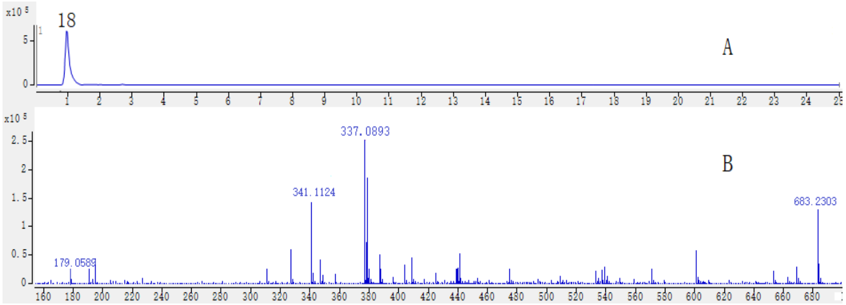

Spectra of Saccharose (18). A: extraction ion chromatography; B: mass spectra in high CE mode.

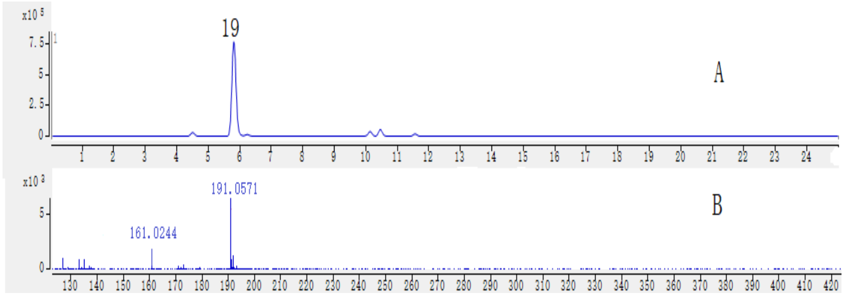

Spectra of Quinic Acid (19). A: extraction ion chromatography; B: mass spectra in high CE mode.

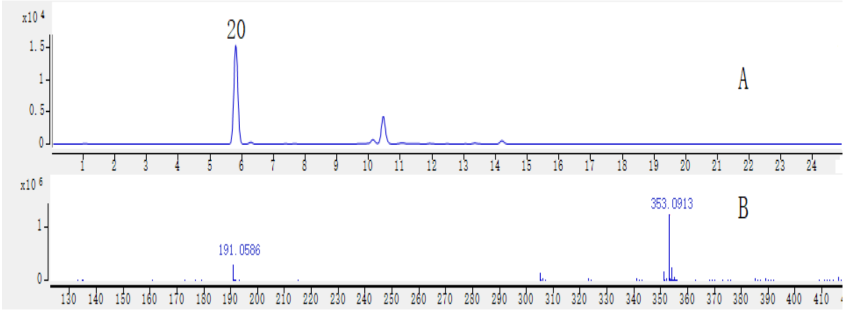

Spectra of Chlorogenic Acid (20). A: extraction ion chromatography; B: mass spectra in high CE mode.

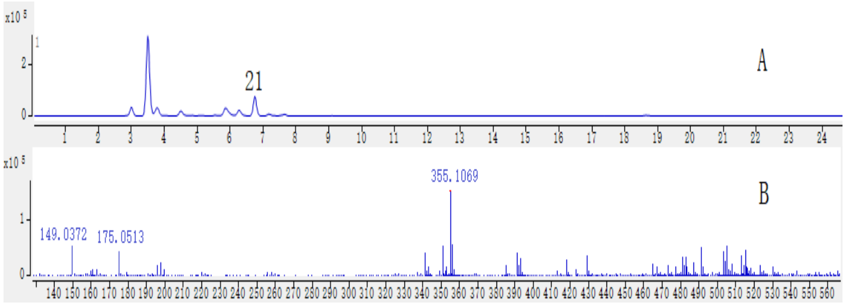

Spectra of Gentiopicrin (21). A: extraction ion chromatography; B: mass spectra in high CE mode.

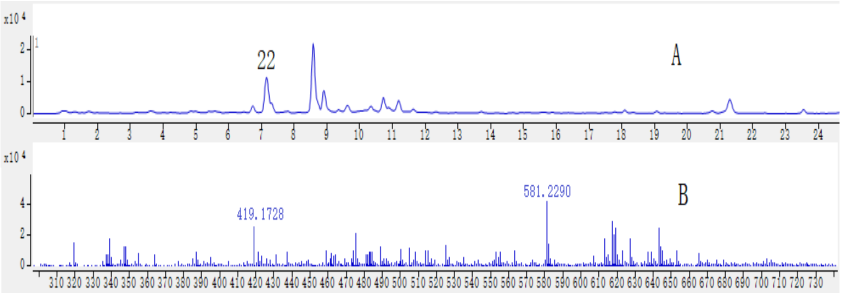

Spectra of 5,5'-Dimethoxylariciresil 4-O-glucoside (22). A: extraction ion chromatography; B: mass spectra in high CE mode.

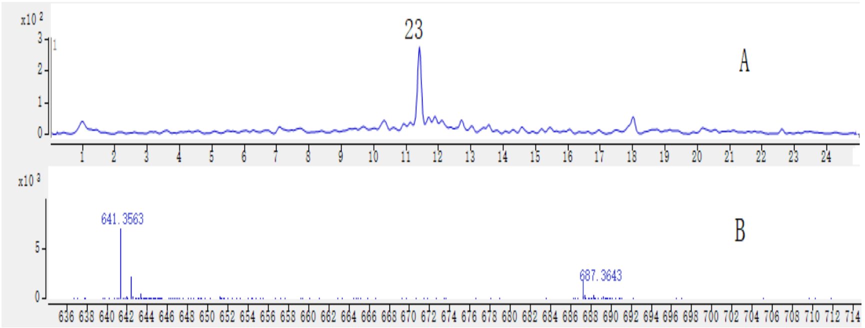

Spectra of Periplocin C (23). A: extraction ion chromatography; B: mass spectra in high CE mode.

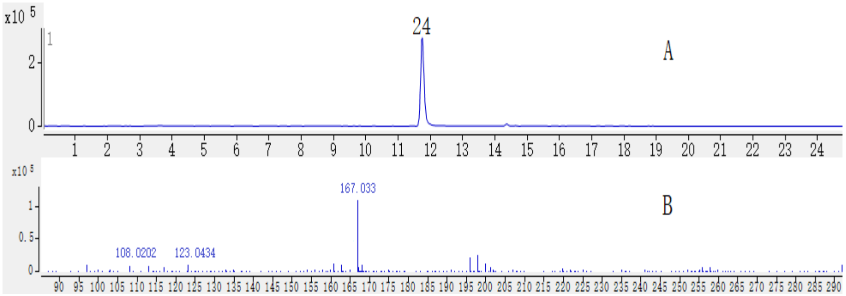

Spectra of 4-Methoxysalicylic acid (24). A: extraction ion chromatography; B: mass spectra in high CE mode.

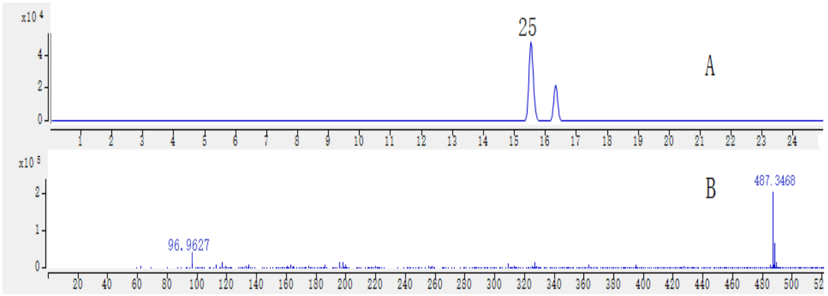

Spectra of Arjunolic Acid (25). A: extraction ion chromatography; B: mass spectra in high CE mode.

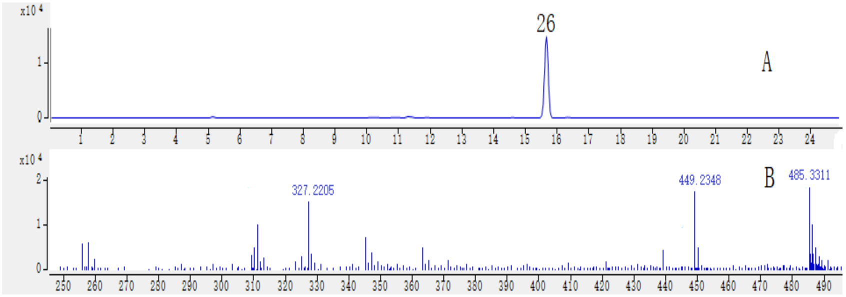

Spectra of 24-hydroxyglycyrrhetic acid (26). A: extraction ion chromatography; B: mass spectra in high CE mode.

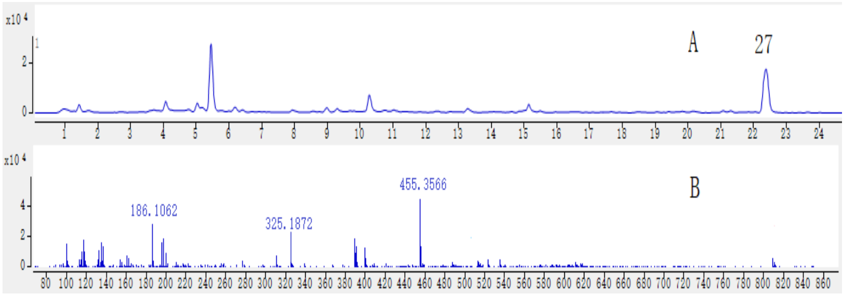

Spectra of Ursolic Acid (27). A: extraction ion chromatography; B: mass spectra in high CE mode.
